# Report on Cholera in 1866

**Published:** 1867-10

**Authors:** H. Wardner

**Affiliations:** Chairman of Committee


ARTICLE XLIII.

### REPORT ON CHOLERA IN 1866.

By H. WARDNER, M.D., Chairman of Committee.

Read before the Alexander County Medical Society.

To the Alexander County Medical Society,

*Gentlemen:*—Your Committee appointed to investigate the subject of cholera, and to report on its character, progress, and prevalence in the City of Cairo and vicinity, beg leave to submit the following, as the result of their labors:—

The disease first made its appearance about the first of August. The first case which attracted special notice, occurred on one of the wharf-boats on the 4th, and from this date to the 15th, the disease increased rapidly and was, comparatively, quite fatal. It prevailed especially on Commercial Avenue, between Second and Sixth Streets, and in the more filthy localities about the city. The negroes suffered from its ravages much more than the white population.

Immediately after its first appearance, a meeting of physicians was held at the office of Drs. Wardner and Gericke, for the purpose of securing unity of action in the necessary steps to be taken to stay the scourge and drive it from our city.

A committee was appointed to wait upon the City Board of Health, and to urge immediate and efficient regulations in regard to cleanliness, and the sale of unwholesome articles of diet at the markets. The efficiency of the Board in carrying out the suggestions of the Medical Faculty, was, no doubt, the means of arresting the disease and driving it from our midst.

From the 15th of August, it continually abated until the 25th, when it had almost, if not entirely disappeared. Since that date, there have been a few sporadic cases, two or three of which proved fatal.

The total number of deaths from cholera in this city, from its first appearance to the present date, October 9th, a period of seventy days, is *seventy-eight*, giving a fatality of about twenty per cent. This estimate includes all cases of simple and aggravated diarrhœa, cholerine, and cholera-morbus, which, in some degree, always precede an attack of cholera, and, in cholera times, may well be called the first stage of that disease; for gastric and intestinal irritation, whatever be its degree or cause, may prove to be the advance guard which opens the portals, batters down the vital defences, and invites the dread enemy to enter and take possession.

The following case, taken from one of the reports handed to your committee, is here given as a type of the disease, as it appeared in its more active form:—

Mr. ——, aged 35 years, was taken with diarrhœa in the morning. He had eaten freely of cucumbers the evening previous. The diarrhœa continued with increasing severity until 2 o'clock on the following morning, when medical aid was first called in. His condition at that time, is given as follows: Vomiting and purging every few minutes; intense thirst, which he in vain tried to allay by drinking all the water he wanted, but every glass of water swallowed was very soon ejected; every time his bowels moved he would discharge near half a gallon of the rice-water evacuations; his skin was covered with a cold, clammy perspiration; wrist pulseless; legs cold to the knees; arms cold to the shoulders; eyes sunken; nostrils contracted; and lips of a dull leaden hue. He was indifferent in regard to his condition, and although his mind was clear, he had no control over his desire for water, and, unfortunately, his attendants had not the force of character necessary to control him, nor to carry out the physician's orders. In about two hours, he was suffering severely from cramps, which were readily relieved by the application of the following lotion, *viz.*:—

℞. Bisulphide of Carbon,------------------ 1 oz.
Tinct. of Camphor,-------------------- 3 oz.

Mix. Saturate a cloth and apply over the seat of pain.

The vomiting and purging ceased about 8 A.M. A thorough course of stimulants, excitants, and revulsives was persevered in, but all efforts were in vain, death held the vantage ground, and he expired at 6 P.M., thirty-six hours after the attack, perfectly rational to the last moment.

The following report of a case, is taken from the report of another physician, who was called early and saw the patient just one hour from the first of the attack:—

Mr. ——, aged 32, was taken at 11½ P.M., Aug. 13th, and was visited at 12½ A.M. His condition is given as follows, *viz.:* "Active vomiting and purging, with some cramping of the lower extremities; pulse feeble, but not much prostrated. The vomiting and purging first yielded to the treatment, then the cramps shortly ceased, and in about two hours all the symptoms abated, and convalescence was rapid and complete."

The treatment in both cases was active and equally in accordance with the advancement of medical science. They are here given as fair examples of cases which occurred in the practice of every practicing physician of the city; and show, also, the importance of the early attendance of the physician, as a necessary means of saving life.

All the reports of physicians, which your committee had the honor to receive, concur in the statement that the *third stage* of cholera, or collapse, is a term almost synonymous with *death.* Only one or two recoveries have been known to occur from that stage during the prevalence of the disease in our city.

The reports of private physicians show a degree of knowledge and uniformity in treatment, which speaks well for the faculty, in a city of so few literary and professional advantages, and for their interest in their profession and the welfare of their patients; for without this professional zeal and ambition, a practitioner will hardly take the time and trouble to keep pace with the advancement in pathology, therapeutics, and the different branches of the profession, as these reports show their authors to be doing.

The following is an outline of treatment, as gleaned from the different reports:—

*First.* In the primary, or diarrhœa stage, low diet, rest, and anodynes, astringents, and alteratives, combined according to the circumstances of the case, and the judgment of the practitioner. The following has been quite generally used:—

*Recipe.*—Laudanum, one drachm; tinct. of cardamon seeds, fluid extract of rhatany, fluid extract of blackberry root, aromatic syrup of rhubarb, and syrup of ginger, of each half an ounce. Mix. Take a teaspoonful after each discharge until relieved.

*Second.* In the second, or active stage of vomiting and purging, with cramps, the following remedies have been generally used, in combinations suited to each special case, and according to the judgment of the physician in attendance, *viz.*:—

*Recipe.*—Tinct. of capsicum, tinct. of camphor, tinct. of rhubarb, tinct. of opium, and chloroform, the latter being considered as the base of the mixture, given in doses varying from 15 drops to a teaspoonful, repeated as frequently as the circumstances of the case required, from fifteen minutes to one hour or more between doses. It has usually been alternated with, or followed by, alteratives combined with opium.

One practitioner has recommended 10 grains of calomel every hour, as he claims, with good success, and has administered as high as 120 grains before discontinuing the remedy. He considers this a curative treatment. Another practitioner has used a solution of chloride of sodium, in combination with capsicum, or black pepper, in teaspoonful doses every fifteen minutes, with good success. This practice is based on the theory that in the loss of the balance of the vital powers, the blood loses its saline constituents during the active stage of the disease, and that collapse begins with the coagulation of the blood in the capillary vessels, because of the absence of salines, which are necessary to preserve the fluidity of the fibrine and the healthy form and tone of the corpuscles.

Dr. WILLIAMS, in his Principles of Medicine, page 143, referring to cholera, says:—"There can be no doubt, therefore, that the blood coagulates in the vessels for want of saline matter, and the red corpuscles become dissolved and altered."

The authorities inform us that the injection of saline solutions into the veins of choleric patients in collapse produces a renewal of life, a return of heat, healthy color of the skin, etc., but that the discharges will often return, and the disease will terminate fatally. Why? May it not be because in the loss of the equilibrium of the fluids, the exosmose predominating and the mucous membrane of the intestines being in a state of irritation, the coats of the capillary vessels relaxed, and, consequently, *receiving* too large of a share of the revived circulating fluid without the power of contraction and propulsion, the loss of serum very soon leaves the blood in its previous stagnant condition?

Now, it is believed that the administration of the saline remedies, especially during the active stage of cholera, lessens the exudation of the fluids of the blood, and tends to restore the equilibrium between the endosmose and exosmose; and the excitant given in combination with it assists in reëstablishing the nervous susceptibility and the healthy absorption of fluids by the capillaries of the stomach and *primæ viæ*. But, this not being the proper place for a lengthy argument in favor of *any* particular theory or treatment, we only add that the practice is claimed to be very successful, and a more consistent manner of using remedies than by forcing them into the circulation with a syringe.

Another practitioner has suggested, and employed in his treatment, the officinal solution of chloride of calcium, in conjunction with the usual remedies, as before described. This practice is based on the theory of decomposition of the blood, and is considered efficient in counteracting the deleterious influence of gases and other poisonous substances resulting from such decomposition. We consider this suggestion worthy of further trial. We know it to be a valuable agent in low and putrid fevers, and would like to hear or read an argument in favor of its use in cholera.

Externally, free use has been made of artificial heat, friction, counter-irritation, etc. Among the more efficient means for allaying the cramps, we find the application of chloroform on porous paper covered with oiled-silk, to prevent evaporation, a valuable remedy. If the surface has been previously irritated by the use of mustard, the chloroform will take effect much more speedily. The lotion composed of bisulphide of carbon and tincture of camphor, applied to the parts affected with cramps, will afford immediate relief. Disinfectants should be continually used in the sick room, and the cholera discharges destroyed by chemical agents at once. In addition to the foregoing outline of treatment, the strictest watch must be kept over the patient, to prevent him from indulging his uncontrollable thirst for cold water. Unless this precaution be heeded, the most skilful treatment will be very liable to prove useless.

The disease having abated, great care must be taken in the patient's diet during convalescence. At first, nourishing drinks, given in small quantities and frequently repeated, together with animal broths and extract of beef well salted, together with such mild medicinal agents as may be deemed proper by the attending physician, will shortly restore the patient to health.

The reports furnished your committee do not exhibit the difference in the prevalence of the disease between the white and black races, but from casual observation it is estimated that in every five cases, three occurred amongst negroes. Amongst the whites, it prevailed, with few exceptions, where the poor and destitute were congregated, and especially in the more filthy localities. The Sewell House and its neighborhood is cited as an illustration. The cistern in use at that house was within five feet of a filthy privy vault, which had so contaminated the water that the Board of Health reported that they observed but little difference in the odors arising from either. The free use of this filthy water is believed to have been the cause of the great severity and fatality of the disease in that locality. It has been suggested that the use of water from filthy cisterns has given rise to many cases of the disease. Almost every case could be easily and distinctly traced to the exciting cause, which was very frequently found to be overindulgence in the use of green vegetables. Melons have been accused of producing more cases than any other article of that class. Cucumbers, cabbage, green corn, and new potatoes all come in for a share of blame.

This community may well commend the wisdom that forbade the sale or giving away of those articles of diet for a limited time, and that ordered the free use of disinfectants wherever offensive and deleterious gases were discovered; for there can be no doubt, in the minds of reasoning people, that the action of the city authorities, in that instance, was the blow that held the monster at bay and drove him from our midst. We verily believe that but for this course, he would be a terror in our city to-day.

We say to those young men of our city who organized themselves into a society for the relief and assistance of the destitute and homeless, and who by personal and pecuniary sacrifices, during the stay of the epidemic, aided many a poor sufferer, that their efforts were not in vain, and that the gratitude of the helpless is not their only reward. The memory of their noble conduct will live in the minds of this community, and be rehearsed in their praise, when they "shall have been gathered to their fathers."

In conclusion, a word about Cairo, and we have done. This city, which has borne all the opprobrious epithets of which the English language is capable, has proved herself victorious in a conflict from which Cincinnati, Louisville, St. Louis, Nashville, and Memphis have come out second best. Cairo is triumphant, and is, to-day, as healthy a city as there is in the United States. Many arguments, as well as statistics, could be brought to prove this assertion. Several physicians have sought other localities, where there is a greater demand for medical services. Those remaining, make their daily rounds in a slow, easy pace, conscious that their gratuitous advice and suggestions in sanitary matters have been, and still are, of incalculable benefit to this community, and a serious detriment to their own pockets.

